# Porous Titanium Granules in comparison with Autogenous Bone Graft in Femoral Osseous Defects: A Histomorphometric Study of Bone Regeneration and Osseointegration in Rabbits

**DOI:** 10.1155/2019/8105351

**Published:** 2019-12-14

**Authors:** Eudoxie Pepelassi, Despina Perrea, Ismene Dontas, Christian Ulm, Ioannis Vrotsos, Stefan Tangl

**Affiliations:** ^1^Department of Periodontology, School of Dentistry, National and Kapodistrian University of Athens, Athens, Greece; ^2^Laboratory for Experimental Surgery & Surgical Research “N. S. Christeas” School of Medicine, National and Kapodistrian University of Athens, Athens, Greece; ^3^Department of Oral Surgery, University Clinic of Dentistry, Medical University of Vienna, Vienna, Austria; ^4^Austrian Cluster for Tissue Regeneration, Vienna, Austria; ^5^Karl Donath Laboratory for Hard Tissue and Biomaterial Research, Department of Oral Surgery, University Clinic of Dentistry, Medical University of Vienna, Vienna, Austria

## Abstract

**Background:**

The high resorption rate of autogenous bone is a well-documented phenomenon that can lead to insufficient bone quality and quantity in an augmented area. Nonresorbable bone substitutes might perform better than autogenous bone in certain applications if they are able to provide adequate bone formation and graft osseointegration.

**Purpose:**

The aim of this study was to compare the osseous regeneration and graft integration in standardized defects in the rabbit femur treated either with porous titanium granules or autogenous osseous graft.

**Materials and Methods:**

Standardized femoral osseous defects were surgically induced in 45 New Zealand rabbits. Fifteen were treated with porous titanium granules (TIGRAN™-PTG) and membrane (PTGM), 15 with autogenous graft and membrane (AGM), and 15 with membrane alone (CM, control). At six weeks, the defects were assessed histologically and histomorphometrically.

**Results:**

PTGM as compared to AGM presented similar percentages of newly formed bone tissue, but a significantly higher fraction of the region of interest was filled with the bone substitute material. Accordingly, the composite of new bone plus bone substitute material showed significantly higher volumes for PTGM. Yet, the smaller amount of remaining autogenous bone was far better osseointegrated than the titanium granules, which in large regions showed no connection to newly formed bone. Both PTGM and AGM as compared to CM presented higher values of newly formed bone.

**Conclusions:**

This study demonstrated that PTG was similarly effective as autogenous osseous graft in achieving osseous regeneration while PTG performed markedly better in graft volume stability. The resulting higher total percentage of new bone combined with the bone substitute material in PTG could provide a superior foundation for implant placement.

## 1. Introduction

Autogenous osseous grafts are often considered ideal for osseous regeneration. However, their use presents limitations, such as high morbidity to the donor site, limited availability, and relatively high and unpredictable resorption [1].

Alloplastic grafts are natural or synthetic materials that function as bone substitutes. Among the alloplastic grafts, few possess osteoconductive properties, whereas many of them act as space fillers. A bone substitute, when grafted in an osseous defect, should provide a proper environment for new bone formation and maintain the space where new bone could grow in [1]. There is increasing demand for alloplastic graft materials in Implant Dentistry. Porous titanium granules (PTGs) are a biocompatible, nonresorbable alloplastic graft material with osteoconductive properties [2–11]. They are commercially available as Natix® (Tigran Technologies AB, Malmö, Sweden) in metallic (PTG) or oxidized (white, WPTG) form and are prepared as irregularly shaped and highly porous granules of 0.7–1.0 mm diameter and with a total titanium surface of each granule close to 2 cm^2^, as assessed by the manufacturer [5]. PTG was considered to be made of commercially pure titanium (cpTi) [5]. However, PTG surface was recently found to have Ti (90.06 ± 11.34%) as well as elements of Na (8.88 ± 9.98%), Cl (2.44 ± 1.96%), and Al (0.99 ± 0.37%), when assessed at ×5000 magnification [12].

Porous titanium granules were first used in Orthopedics [2, 3] and then began to be applied in Dentistry [4, 13, 14]. Porous titanium granules have been studied in postextraction sockets [7, 15–17], maxillary sinus augmentation [4, 6, 18–21], peri-implant defects [9, 10, 22–26], supracrestal vertical bone augmentation [27], and periodontal defects [23, 28]. They have been studied in various animal models, such as rabbits [5, 6, 8, 27], dogs [3, 7, 9, 15, 16], sheep [11], and mini-pigs [10, 17, 28], and humans as well [4, 13, 14, 18, 19, 21–26, 29, 30]. Among all animal models for medical and dental research, rabbits are widely used [31–33].

PTG has been studied alone or in combination with xenograft [20]. Porous titanium granules have been compared to demineralized bovine bone mineral (DBBM) in animals [9, 10, 28], to bovine hydroxyapatite in animals [6], and to deproteinized bovine particulates (Bio-Oss) in humans [19]. Comparisons between PTG (or WPTG) and autogenous osseous graft, which is the clinical gold standard, have not been published yet. Such a study would provide important information because autogenous bone has a known propensity to be resorbed quickly and shows poor volume stability. Titanium granules on the other hand are nonresorbable and could therefore perform better in clinical situations where high bone resorption rates prevail [34, 35]. This led the authors to examine the hypothesis that the osseous regeneration in experimental animal osseous defects is different between defects grafted with PTG and defects grafted with autogenous osseous graft.

The aim of the present study was the histological and histomorphometric comparison of the osseous regeneration and the graft integration in experimentally induced osseous defects in the rabbit femur treated with porous titanium granules or autogenous osseous graft.

## 2. Materials and Methods

### 2.1. Animals, Anaesthesia, and Surgery

Forty-five New Zealand white male rabbits (3.5–4 kg) were used. The animals were randomly assigned to three groups of 15 each: (1) treatment of the surgically induced femoral osseous defect with porous titanium granules (TIGRAN™-PTG) and resorbable collagen membrane (PTGM), (2) treatment of the surgically induced femoral osseous defect with autogenous osseous graft and resorbable collagen membrane (AGM), and (3) treatment of the surgically induced femoral osseous defect with resorbable collagen membrane alone (CM or control). They were acclimatized to the experimental conditions for one week prior to the study initiation.

The animals were housed one per cage in stainless steel wire net cages, fed a standard rodent diet with free access to water, and exposed to a 12 h light/dark cycle, at room temperature 18–22°C and relative humidity 55–65%. All animals were kept in their allocated cages for the entire study duration. The study was conducted in accordance with guidelines approved by the Council of the American Psychological Society (1980), the European Communities Council Directive of 24 November 1996 (86/609/EEC), and the Hellenic Presidential Decree 160/91. The study was performed meeting ARRIVE guidelines and approved by the University of Athens Ethics and Research Committee (Ref. 167/12.05.2011) and by the Veterinary Directorate of the Prefecture of Athens.

On day one of the study, the femoral osseous defect was surgically induced and treated with the allocated treatment for each animal. For premedication, 25 mg/kg ketamine hydrochloride (Ketaset, Ceva) and 5 mg/kg xylazine (Rompun, Bayer) were administered intramuscularly. Following premedication, 4 mg/kg carprofen (Rimadyl, Pfizer) and 5 mg/kg enrofloxacin (Baytril, Bayer) were administered subcutaneously for analgesia and antimicrobial prophylaxis, respectively. Additionally, thiopental sodium (Pentothal, Abbott) was administered intravenously, through a 21 G butterfly catheter placed in the marginal ear vein, in N/S 0.9% (10 ml/kg), for maintaining general anaesthesia. The animal was intubated with a 3.0 mm ID cuffed endotracheal tube (Mallinckrodt) and attached to a small animal ventilator (Harvard Apparatus model 683). The animal was monitored during surgery with pulse oxymetry (Kontron Instruments Pulse Oximeter 7840) and blood pressure monitoring (Dinamap-Criticon Vital Signs Monitor 1840). The right lateral femoral condyle was depilated, disinfected, and covered with a sterile drape. The incision was made, and the flap was elevated at the predetermined site of the right femur. Then, an osseous defect, 6 mm in diameter [36] and 5 mm in depth, was induced in the distal metaphyseal-diaphyseal area by using a trephine surgical drill of specific diameter at a low speed under copious saline irrigation. Then, the defect was either left empty for the CM group or completely filled (up to the level of the original cortical surface of the femur) with PTG particles for the PTGM group or with autogenous osseous graft for the AGM group. The latter two groups therefore equally received a volume of substitute material that corresponds to the volume of the standardized drill hole. Each defect was covered in all groups (PTGM, AGM, and CM) with a resorbable collagen membrane (Geistlich Bio-Gide®, Wolhusen, Switzerland), which was secured in place by using nonresorbing titanium screws, 1.3 mm wide and 4 mm long (stoma ossecure®, Storz am Mark GmbH, Emm.-Liptingen, Germany). For the AGM group, the autogenous osseous graft was obtained from the corresponding site of the left femur. For this purpose, the same procedure was followed regarding surgical site preparation, incision, and flap elevation. A hollow trephine was used to harvest the osseous graft, which was then crushed in particles with the help of an osteotome and mixed with blood. The osseous defect of the right femur was filled with the graft particles, originating from the left femur. In order to create the same experimental conditions for all animal groups, the same procedure was followed for PTGM and CM groups without using the harvested graft. Finally the flap was repositioned and sutured. Upon surgery completion, each rabbit was placed on a heating blanket to recover from anesthesia without the risk of hypothermia and then returned to its allocated cage. Carprofen and enrofloxacin were administered postoperatively for seven days. Postoperatively, both surgical sites were daily inspected and the animal was under the care of the laboratory's veterinarian. Sutures were removed at seven days. Standard presurgical, surgical, and postsurgical conditions were kept for all animals. At six weeks, each animal was euthanized to harvest the right femur. Prior to euthanasia, all surgical sites were visually inspected to evaluate wound healing and detect possible complications. For the euthanasia, ketamine and xylazine intramuscular premedication was administered, as previously described, and followed by 50 mg/kg thiopental sodium intravenous infusion in N/S 0.9% through the marginal ear vein.

### 2.2. Histological and Histomorphometric Assessment

Immediately after euthanasia, the right femur was dissected and bone blocks containing the area of the osseous defect were removed. After fixation in buffered 4% formaldehyde solution [37] for at least 2 weeks, bone blocks were dehydrated in ascending grades of alcohol and embedded in Technovit 7200 (BPO Kulzer & Co, Werheim, Germany). Bone blocks were further processed according to the Cutting and Grinding technique described by Donath [38]. Fifty *μ*m thick undecalcified thin-ground sections, precisely through the long axis of the defect, perpendicular to the long axis of the femoral diaphysis, were prepared and stained with Levai–Laczko dye [39]. This standard dye allowed for the reliable differentiation between old and new bone tissue, bone substitute, and soft tissue due to their distinct affinities for the stain.

Stained sections were photographed with the Olympus Dot Slide system 2.4 (Olympus, Tokyo, Japan), resulting in overview images with a resolution of 2.5726 *μ*m per pixel. These digitized images provided the basis for histomorphometric analysis: A rule set for the histomorphometry software Definiens Developer 7 (Definiens, Munich, Germany) was devised considering the color and shape of the objects themselves, as well as the relationship to other neighboring objects in contact with them. By this means, the tissue types, PTG, AG, newly formed bone tissue, and marrow area/soft tissue, were automatically segmented and classified. The few falsely classified areas were manually corrected under visual control using Adobe Photoshop CS3 (Adobe, San Jose, CA, USA).

**Figure 1:**
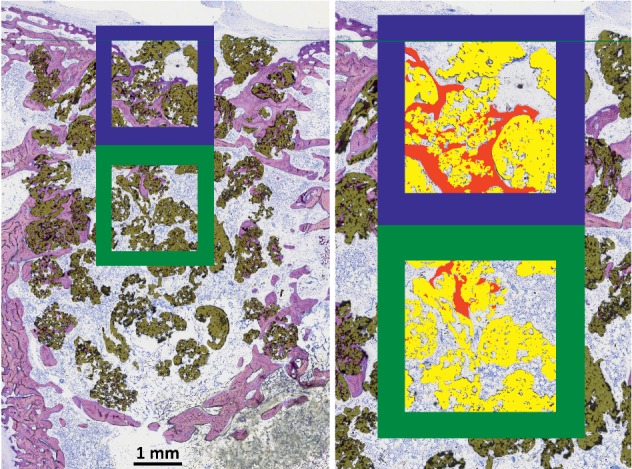
Positions of the regions of interest (ROIs) for the histomorphometric evaluation. A blue frame surrounds the cortical area and a green frame the medullary ROI. On the right side, classified newly formed bone is depicted in red and bone substitute material in yellow.

In order to gather information about the quantity of bone regeneration within the defect, the percentage of newly formed bone within the complete region of interest (volume of newly formed bone per tissue volume; nBV/TV) was assessed. The amount of the bone substitute in the defect area (bone substitute volume per tissue volume; BSV/TV) was calculated to characterize the volume stability and packing density of the grafted materials. As new bone tissue can only be laid down in areas where there is no bone substitute material (PTG or AGM) present, the size of this available space between the particles has great influence on the amount of bone neoformation. Therefore, the percentage of newly formed bone in the available space between the bone substitute materials (newly formed bone per available volume; nBV/Av.V) was also calculated [40]. To characterize the total amount of material which is potentially biomechanically active, the percentage of newly formed bone plus bone substitute material in the region of interest (composite volume per tissue volume; Co.V/TV) was determined [40]. As a measure of the quality of integration of grafted materials, the percentage of bone substitute surface that was in contact with newly formed bone (bone-to-bone-substitute contact; BBSC) was quantified. All these histomorphometric evaluations were performed in two separate regions of interest: one located in the cortical area of the defect where the opening of the drill hole had been and the other immediately underneath the first, deeper in the medullary compartment (Figure 1). Additionally, the percentage of bone substitute particles that were in contact with newly formed bone tissue in at least one spot of their contour was determined (particle integration rate: PIR) In this case, all of the particles visible in the histologic specimens were included in the evaluation.

### 2.3. Statistical Analysis

For variables nBV/TV, nBV/Av.V, Co.V/TV, BS.V/TV, and BBSC, descriptive statistics (mean, median, standard deviation (SD), interquartile range (IQR), minimum, and maximum) and boxplots were created. In order to achieve approximate normal distribution, the natural logarithm of nBV/TV, nBV/Av.V, Co.V/TV, and BS.V/TV was used, residuals were checked graphically. Models included area and treatment as independent variables and ID as the random variable. ANOVA was calculated to test for the influence of treatment. Post hoc Tukey tests were performed. As secondary hypotheses, the medullary and cortical areas were tested. Fisher–Pitman permutation tests were calculated for BBSC and PIR. *p* values were adjusted for multiple testing using the Benjamini–Hochberg method [41]. All calculations were performed with R 3.5.3 software [42], and ggplot2 [43] was used for creating graphics.

## 3. Results

Nine animals (three in each group) died of anesthetic complications or postsurgical infection before they could finish the scheduled duration of six weeks and had for this reason to be excluded from the study. They were replaced by nine new animals which finished the planned course of experimentation. Therefore, 45 animals (15 in each group) in total completed the experiment and were finally studied. The survival rate was 83.33% for each animal group.

### 3.1. Morphological and Qualitative Description

**Figure 2:**
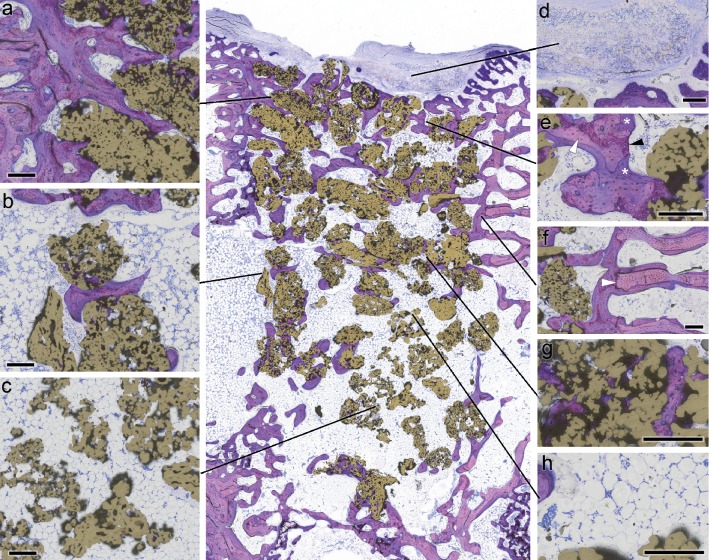
Histological characterization of PTG osseointegration. In the center, the distribution of PTGs (gold-colored) within the drill hole is visible in an overview image. (a) Detail of the strong osseointegration in the cortical compartment. (b) Much weaker osseointegration in the medullary region. (c) In large areas, the PTGs showed no signs of osseointegration at all. (d) Remnants of the resorbable membrane were still detectable. (e) Newly formed bone tissue consisted mostly of woven bone (black arrowhead) compacted by parallel-fibred bone (white arrowhead). This primary bone had already been partly remodeled into secondary lamellar bone (asterisk). (f) Border of the drill hole (white arrowhead). (g) Newly formed bone tissue was laid down in the pores of the PTGs. (h) The spaces between the granules were mostly filled with fatty marrow (microphotograph of undecalcified thin‐ground section; Levai–Laczko stained; length of scale bar equals 200 *μ*m).

After six weeks, the old autochthonous and newly formed bones were readily distinguishable (Figures 2(a) and 2(f)). Old bone that had been present before the defect was drilled appeared in a light pink hue while the bone tissue that was formed during the healing process was stained in dark purple. On the basis of these criteria, it was possible to recognize and reconstruct the area and borders of the original drill holes.

**Figure 3:**
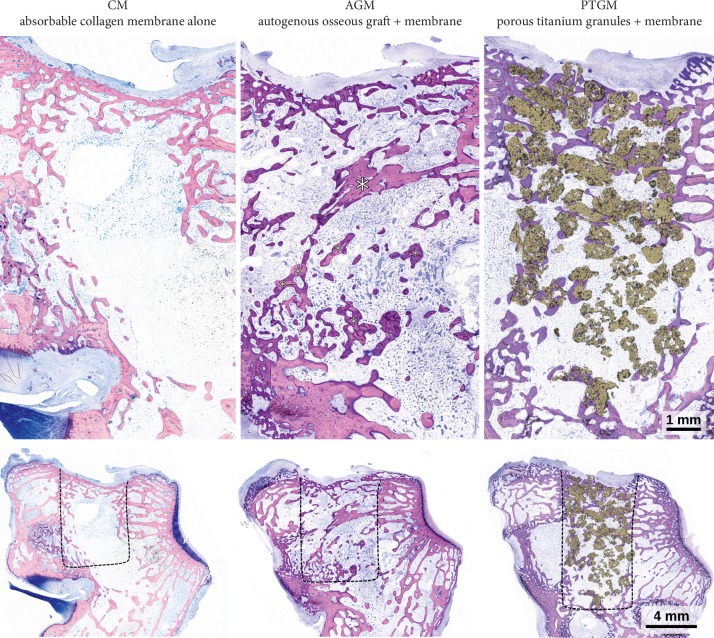
Comparison of defect healing and histological osseointegration in the three treatment groups (views of complete defects above and overview images of the lateral condyle below). The control group (resorbable membrane alone) showed very little new bone tissue in the marrow compartment of the defect. Bone formation was clearly stronger in the group treated with autogenous bone. Osseointegrated remnants of the graft (asterisks) were still present. In the defect filled with PTG, many titanium granules were detectable and well integrated into new bone tissue. The composite of granules and surrounding bone fills the defect to a larger extent than is the case in the other groups. Black dashed lines indicate the borders of the drill holes (microphotograph of horizontal undecalcified thin‐ground sections, Levai–Laczko stained).

All three treatment groups had healed well, granules were evenly distributed inside of the drill holes, and no strong displacements into the surrounding tissues were observed, but there were differences in the amount of regenerated bone tissue and also in its spatial distribution. In general, the strongest bone formation took place in the region of the aperture in the cortical bone (Figure 2(a)). However, this area was characterized by a depression (Figure 2(d)) that had the geometry of a shallow bowl. These structures were caused by the fact that the covering membrane which was still detectable (Figure 2(d)) bulged into the drill holes, thereby prohibiting bone formation in the depressed area. This phenomenon was much stronger in the control group than in the AGM and especially than in the PTGM group, where membrane prolapse was reduced by the presence of grafting material. Underneath the membrane, the opening of the drill hole was in most cases closed by a network of woven bone that had been compacted with parallel-fibred bone tissue and partly transformed by secondary remodeling into lamellar bone (Figure 2(e)). Particles of the autogenous bone graft or titanium granules, respectively, were incorporated into this newly formed bone (Figure 3). Some PTGs partly protruded into the soft tissue space (Figure 2 center) or even lay completely detached outside of the cortical bone (Figure 1) and were surrounded by fibrous tissue.

In the deeper regions of the former drill hole, the differences were more pronounced. While in the CM group, these areas appeared to be almost free of bone tissue or were bridged by sparse cancellous trabeculae (Figure 3); in the PTGM group, large amounts of loosely packed granules were present (Figure 2). In the periphery of the drill hole, close to the old autochthonous bone, the particles were incorporated in and surrounded by new bone (Figure 2(b)), while in the central regions, the histological osseointegration was far less pronounced (Figure 2(c)). Here, many PTGs exhibited no bone formation on their surfaces at all. There was an obvious gradient in the intensity of bone formation from the margins towards the center of the defect. Concerning these matters, the AGM-treated animals lay somewhere in between the other treatment groups (Figure 3). For the AGM group, more bone and cancellous trabeculae were present than in the CM animals but there remained only very few osseointegrated remnants of the autogenous bone transplant. The surfaces of these remaining particles were almost completely covered by newly formed bone. The combined amount of newly formed bone plus bone substitute was therefore much lower for AGM than PTGM where large amounts of titanium granules filled the defect.

There were no detectable differences in vascularization, tissue maturity, or the structure of the nonmineralized tissues in the test area, which predominantly consisted of fatty marrow (Figure 2(h)). No strong signs of resorptive processes or inflammations could be observed in either group, and no multinucleated giant cells were present on the surface of the PTG particles (Figure 2(h)).

### 3.2. Histomorphometric Findings

**Figure 4:**
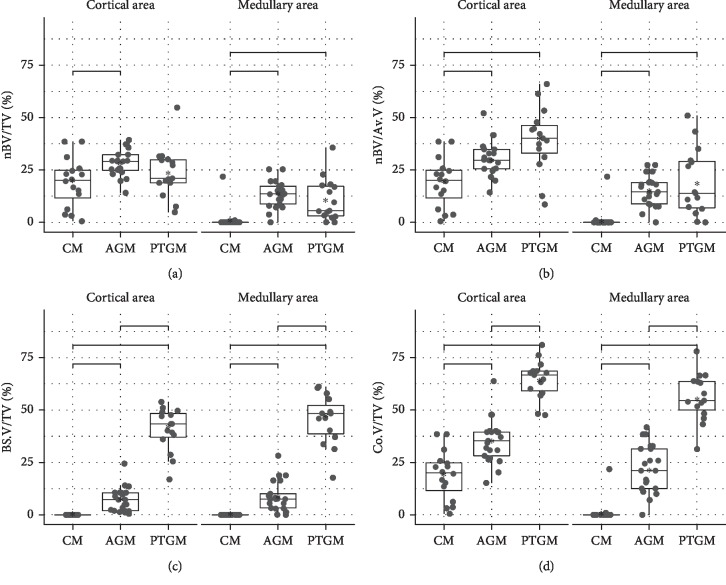
Histomorphometric results for volumetric data: (a) percentage of newly formed bone tissue in the whole region of interest; (b) percentage of newly formed bone in the spaces available in between the bone substitute particles; (c) percentage of bone substitute material in the region of interest; (d) percentage of the composite consisting of newly formed bone plus bone substitute material in the region of interest.

**Figure 5:**
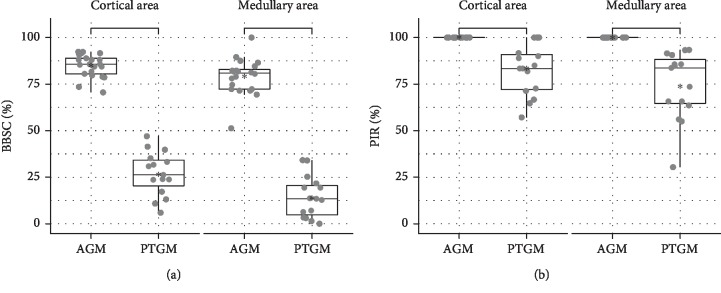
Histomorphometric results concerning osseointegration: (a) percentage of the surface of the bone substitute particles that is in contact with newly formed bone in the region of interest; (b) percentage of the bone substitute particles that have at least one contact to the newly formed bone in the complete defect area.

Descriptive statistics for nBV/TV, nBV/Av.V, BS.V/TV, Co.V/TV, BBSC, and PIR in the cortical and medullary areas are presented in Table 1, respectively. Post hoc tests for the comparisons among treatment groups are presented in Table 2. Boxplots for the variables studied are presented in Figures 4 and 5. Comparison between PTGM and CM revealed that there were statistically significant differences for all parameters in the medullary and cortical areas, except for nBV/TV in the cortical area (Table 2). Specifically, all mean values were statistically significantly higher for PTGM than CM, except for nBV/TV in the cortical area which was nonstatistically significantly higher for PTGM than CM (Tables 1 and 2). Comparison between AGM and CM revealed that there were statistically significant differences for all parameters both in the cortical and medullary areas (Table 2). Specifically, all mean values were statistically significantly higher for AGM than CM (Tables 1 and 2). Comparison between PTGM and AGM revealed both in the cortical and medullary areas absence of a statistically significant difference for nBV/TV and nBV/Av.V and existence of a statistically significant difference for Co.V/TV and BS.V/TV (Table 2). Specifically, nBV/TV was nonstatistically significantly lower for PTGM than AGM both in the cortical and medullary areas, nBV/Av.V was nonstatistically significantly higher for PTGM than AGM both in the cortical and medullary areas, and Co.V/TV and BS.V/TV were statistically significantly higher for PTGM than AGM both in the cortical and medullary areas (Tables 1 and 2). BBSC and PIR were significantly lower for PTGM than AGM both in the cortical and medullary areas (Table 1).

For nBV/TV, there was a statistically significant difference in the comparisons of PTGM and AGM to CM in the medullary area and in the comparison between AGM and CM in the cortical area (Table 2). Mean nBV/TV value was highest for AGM and lowest for CM both for cortical and medullary areas (Table 1).

For nBV/Av.V, comparisons of PTGM and AGM to CM presented statistically significant differences, whereas the comparison between PTGM and AGM did not show statistical significance both in the cortical and medullary areas (Table 2). Mean nBV/Av.V value was highest for PTGM and lowest for CM both for cortical and medullary areas (Table 1).

For Co.V/TV, comparisons between the groups presented statistically significant differences both in the cortical and medullary areas (Table 2), with PTGM having the highest mean values and CM the lowest (Table 1).

The possible influence of the treatment on nBV/TV, nBV/Av.V, and Co.V/TV was studied by one-way ANOVAs, for medullary and cortical areas separately. For cortical areas, the *p* value was 0.0224 for nBV/TV and <0.001 for the rest of the variables. For medullary areas, all *p* values were <0.001.

Then, the possible different behavior of the variables nBV/TV, nBV/Av.V, and Co.V/TV in the medullary and cortical area was studied. For the hypothesis that the medullary and cortical areas did not differ in effect, the *p* values for nBV/TV, nBV/Av.V, and Co.V/TV were <0.001.

## 4. Discussion

The present study investigated the osseous regeneration and graft integration in well-established standardized defects in the rabbit femur [33] treated either with porous titanium granules or autogenous osseous graft. All defects were covered with resorbable collagen membranes to prevent soft tissue invasion and graft dislocation [8, 31]. New bone formation, integration of bone graft particles, and graft volume stability were histomorphometrically and histologically analyzed.

In summary, osseous regeneration was comparably strong in the AGM and the PTGM group but significantly lower in the CM group. PTGM showed significantly higher graft volume stability but weaker osseointegration than AGM. Bone regeneration turned out to be highly associated with the stability of the grafting materials used.

In detail, PTGM exhibited a lower percentage of newly formed bone in the total region of interest than AGM. However, nBV in the available volume (i.e., the space that is not occupied by bone graft) was higher than in the AGM group. This paradoxical phenomenon can be explained by the fact that autogenous bone undergoes extensive resorption leading to the reduction of the graft volume as reported in various studies [44–51]. Consequently, the available “empty” volume (unoccupied by bone graft particles) increases over time and thereby the volume where new bone can grow into. Porous titanium particles on the other side are nonresorbable and remain stable in volume over time. Thus, in PTG-treated defects, the volume where new bone can be formed does not increase in the course of time.

In other words, at the moment of grafting, tissue volume was the same for both groups since all drill holes had the same standardized dimensions. In the AGM group, new bone could continuously replace the resorbed autograft in an increasing available space. Within the constant available space between the titanium granules, this group performed as well as AGM. The osseous regeneration thus might be regarded as similarly effective in PTGM as compared to AGM.

No foreign body reaction was observed in either group. In addition to this good biocompatibility [19] and the osteoconductive properties of PTG [2–11, 15, 29], the volume stability over time, the stable packing density, and the porous microstructure make PTG a clinically relevant biomaterial. PTG particles act as a scaffold and space holder for new bone invasion without being subjected to a time-dependent degradation. The PTG structure allows bone apposition around, in-between, and inside the porous particles [15, 18, 19, 52]. New bone was formed in the larger pores of the PTG particles but not in the smaller ones. This is in agreement with previous findings [18, 53, 54]. The most likely reason for this is that osteoblasts are too large to penetrate the smaller pores and therefore cannot form bone in the deeper regions of the granules. A light packing of the PTG particles is preferable, since compression reduces the dimensions of the interparticular spaces, thereby also reducing the available room for bone apposition [53].

From a clinical point of view, the volume of the newly formed bone and the volume of the graft material together might be regarded as a biomechanically relevant entity. In oral implantology and orthopedic surgery, the osseointegrated bone graft serves as a physical foundation for the insertion of endosseous implants.

In this study, concerning the amount of “materials” that are biomechanically relevant (new bone plus bone substitute), PTGM was superior to AGM since these values were significantly higher for PTGM. In case of implant placement, the presence of a larger volume of “biomechanically relevant materials” should theoretically provide better support and stability for implants.

However, these positive findings in the PTGM group were somewhat diminished by the weaker osseointegration of the titanium granules. Bone-to-bone-substitute contact was lower for PTGM than AGM in both the cortical and medullary areas. In fact, a significant percentage (about 25%) of the PTG particles was not in contact with the newly formed bone at all while literally every piece of remaining, unresorbed autogenous bone showed the presence of some new bone on its surface. This proves that autogenous bone was better osseointegrated than PTG at six weeks. Such a lower bone-to-bone-substitute contact was found earlier for PTG as compared to xenograft at six months after maxillary sinus augmentation in rabbits [6].

Only when the bone substitute material is in direct contact with the network of newly formed bone, mechanical forces can be transferred from the implant to its osseous environment. A tighter connection between graft and newly formed bone therefore should ensure improved stability. In this aspect, PTG appears biomechanically inferior to autogenous graft, since a significant portion of the PTG particles can probably not contribute to provide clinically relevant mechanical stability.

Combining the findings on composite volume and on bone-to-bone-substitute contact, the present study showed a contradictory picture. PTG, as compared to autogenous graft, provided a significantly larger volume of materials for potential mechanical support at six weeks but actually achieved less contact with the newly formed bone and thus only undeterminable biomechanical potential. These considerations suggest that a larger amount of material available for osseointegration, which might be thought to offer a more stable environment for implant placement, does not necessarily result in better biomechanical stability. From the data at hand it cannot be deduced of which practical relevance these differences are. Only biomechanical testing of implants placed in areas augmented with the two materials studied here could answer this question.

It is legitimate to speculate that a closer contact and tighter connection between PTG and newly formed bone might be achieved with time. A possible delay in the histological osseointegration of bone substitute particles cannot be excluded with the use of PTG. Such a delayed healing compared to autogenous bone chips was documented when anorganic bovine bone was filled into extraction sockets [49]. The six-month healing was delayed in PTG as compared to xenograft-augmented maxillary sinuses in animals [6].

Another noteworthy finding was the regional difference of osseous regeneration within the femoral defect: In all groups, less bone was formed in the medullary region than the cortical compartment of the defect. The higher regenerative potential of the cortical region might be attributed to the close local relationship to the periosteum [55, 56] and endosteum [57]. However, also the collagen membrane itself might influence the osseous response in this region: A recent study of Turri et al. [58] demonstrated that naturally derived membranes not only represent a passive barrier but also provide a bioactive microenvironment for pro-osteogenic signals, growth factor, and cells promoting bone regeneration in its close proximity, i.e., the cortical region [58].

In the present study, PTG particles were better integrated in the cortical region than the medullary region of the defect. This suggests the presence of an increased biomechanical stability in the cortical area, a fact that is of practical clinical importance since the critical primary stability of implants is strongly influenced by the amount of cortical bone present [59, 60]. In the medullary area, by contrast, the graft particles were frequently found lying unconnected due to lower integration into newly formed bone. Future studies might elucidate whether the nonintegrated PTG particles in the medullary area could provide additional osteoconductive surface for bone formation at a later stage, for instance, after implant insertion. The trauma connected with this process leads to a second surge of bone formation that could utilize the free surfaces of the granules to lay down new bone tissue, thereby increasing the overall rate of osseointegration.

The lower regenerative potential of the medullary compartment allows conclusions about how bone substitute materials might perform in areas with impaired healing like the deeper regions of a sinus lift [61], while the cortical area can serve as a model for fast, high-powered bone regeneration.

In conclusion, it can be said that in the present study, grafting the osseous defect with PTG was similarly effective in achieving osseous regeneration as with autogenous bone. The graft volume stability of the nonresorbable PTG was clearly far better than that of autogenous bone graft which was quickly resorbed. In total, more biomechanically relevant material, i.e., the combined volumes of newly formed bone and bone substitute material, was present in PTG-treated sites. These results suggest that PTG can equal autogenous bone grafting in facilitating bone formation and surpasses it as a space filler and in providing long-lasting graft stability. These properties might be advantageous for an application in defects where resorption is known to proceed fast such as in extraction sockets.

However, a large percentage of the PTG particles were not in contact with newly formed bone. It cannot be deduced from the results of this study if and to which degree this fact can reduce the mechanical stability of the augmented area. Additional biomechanical testing would be necessary to answer this important question.

## Figures and Tables

**Table 1:** Descriptive statistics for nBV/TV, nBV/Av.V, Co.V/TV, BS.V/TV, BBSC, and PIR per treatment group and anatomical region.

Parameter	Area	Group	Mean	Median	SD	IQR	Min	Max
nBV/TV	Cortical	AGM	27.3	25.4	6.9	7.4	14.1	39.3
		CM	17.7	19.6	10.8	14.0	0.6	38.5
		PTGM	23.8	21.0	12.0	11.0	4.8	54.8
	Medullary	AGM	12.2	13.4	6.3	6.9	0.0	25.3
		CM	1.5	0.0	5.6	0.0	0.0	21.8
		PTGM	10.6	5.5	10.1	14.1	0.0	35.6
nBV/Av.V	Cortical	AGM	29.6	29.5	9.3	8.9	14.3	52.0
		CM	17.7	19.6	10.8	14.0	0.6	38.5
		PTGM	39.4	40.1	15.6	13.1	8.5	66.0
	Medullary	AGM	13.7	14.1	7.4	9.7	0.0	27.3
		CM	1.5	0.0	5.6	0.0	0.0	21.8
		PTGM	18.9	13.9	15.9	22.1	0.0	51.0
Co.V/TV	Cortical	AGM	33.8	31.9	11.8	12.7	15.2	63.8
		CM	17.7	19.6	10.8	14.0	0.6	38.5
		PTGM	64.5	66.7	9.2	9.4	47.7	81.1
	Medullary	AGM	20.7	21.1	11.9	16.9	0.0	41.8
		CM	1.5	0.0	5.6	0.0	0.0	21.8
		PTGM	56.1	54.5	11.4	13.5	31.4	78.0
BS.V/TV	Cortical	AGM	6.5	4.8	6.5	7.8	0.5	24.4
		CM	0.0	0.0	0.0	0.0	0.0	0.0
		PTGM	40.7	43.4	10.4	11.2	16.9	53.9
	Medullary	AGM	8.5	7.5	7.7	6.3	0.0	28.2
		CM	0.0	0.0	0.0	0.0	0.0	0.0
		PTGM	45.5	48.4	11.9	13.5	17.8	61.2
BBSC	Cortical	AGM	84.3	85.4	6.8	9.6	70.6	92.3
		PTGM	26.9	26.3	11.8	13.8	5.9	47.0
	Medullary	AGM	77.8	78.5	11.2	9.7	51.3	100.0
		PTGM	14.3	13.4	11.2	15.7	0.0	34.2
PIR	Cortical	AGM	100.0	100.0	0.0	0.0	100.0	100.0
		PTGM	82.4	83.3	13.5	18.8	57.1	100.0
	Medullary	AGM	100.0	100.0	0.0	0.0	100.0	100.0
		PTGM	74.6	83.7	18.2	23.5	30.4	93.3

Mean and median values, standard deviation (SD), interquartile range (IQR), minimum and maximum values.

**Table 2:** Post hoc tests for the comparisons among the treatment groups.

Area	Group comparison	nBV/TV	nBV/Av.V	Co.V/TV	BS.V/TV	BBSC	PIR
		*p* value	*p* value	*p* value	*p* value	*p* value	*p* value
Cortical area	AGM vs CM	0.024	0.0108	<0.001	<0.001	<0.001	<0.001
	PTGM vs CM	0.197	<0.001	<0.001			
	PTGM vs AGM	0.644	0.6447	0.0083			
Medullary area	AGM vs CM	<0.001	<0.001	<0.001	<0.001	<0.001	<0.001
	PTGM vs CM	<0.001	<0.001	<0.001			
	PTGM vs AGM	0.562	0.9905	<0.001			

## Data Availability

The data used to support the findings of this study are available from the corresponding author upon request.
